# Pre- and post-operative differences between genders in idiopathic macular holes

**DOI:** 10.1186/s12886-020-01633-4

**Published:** 2020-09-10

**Authors:** Jing Wang, Yanping Yu, Xida Liang, Zengyi Wang, Biying Qi, Wu Liu

**Affiliations:** Beijing Tongren Eye Center, Beijing Tongren Hospital, Capital Medical University; Beijing Ophthalmology and Visual Sciences Key Laboratory, No 1, Dongjiaominxiang Street, Dongcheng District, Beijing, 100730 China

**Keywords:** Gender variation, Idiopathic macular hole, Optical coherence tomography, Vitrectomy

## Abstract

**Background:**

To compare idiopathic macular holes (IMHs) between male and female before and after surgery.

**Methods:**

Patients with IMHs of stage 3 and stage 4 who underwent 23-gauge vitrectomy were retrospectively enrolled. Pre-operative clinical features like age of onset, and best-corrected visual acuity (BCVA) were reviewed. Optical coherence tomography parameters including minimum linear diameter (MLD), central macular thickness and some other indexes were measured and calculated. Main surgical outcomes included the primary closure rate, the highest BCVA during follow-up, and the recovery duration. All the metrics mentioned above were compared between genders with appropriate statistical methods.

**Results:**

A total of 298 eyes from 280 patients (male: 51; female: 229) were enrolled. Compared with men, women demonstrated a significantly higher ratio of stage3/stage4 (*P* = 0.045), larger horizontal MLD (*P* = 0.009), but similar surgical outcomes except for a relatively longer recovery duration (*P* = 0.024). For stage 3 IMHs, women exhibited significantly younger age of onset (*P* = 0.023), larger MLD (*P* = 0.003), and smaller height of the hole (*P* = 0.029). However, for stage 4 IMHs, all the pre- and post-operative metrics showed no differences between genders.

**Conclusions:**

Female IMHs seem to demonstrate an earlier age of onset and larger size of hole, especially in IMHs of stage 3. However, these differences, which may owe to normal gender-related variations, have limited influence on the surgical outcomes.

## Background

Idiopathic macular holes (IMHs) are full-thickness defect in the neuroretina with unknown specific causes [1], which greatly reduce vision health-related quality of life [2]. The incidence of IMH is approximately 0.01 to 0.09% [3], with a female to male ratio of about 3:1 [3]. Although the pathogenesis of IMH is not completely clear, vitreomacular traction has been considered as one of the most important motivating factors [4].

Clinically, gender variation is not rare in fundus diseases. Several studies considered male gender as a risk factor for central serous chorioretinopathy (CSC) [5], and female subjects with CSC tend to obtain better prognosis [6]. Another report showed that women had a potentially higher risk of neovascular age-related macular degeneration compared to men [7]. Besides different incidences, male and female IMHs may exhibit different manifestations and surgical outcomes since women demonstrate wider foveal pit and thinner central retina [8]. However, few previous studies have focused on this issue.

This study aims to compare pre-operative clinical features, morphological characteristics (especially OCT parameters) and surgical outcomes of stage 3 and stage 4 IMHs between genders based on a relatively large sample, and try to find differences of IMH between genders so as to provide reference for clinical applications.

## Methods

### Patients and examinations

Patients with IMHs of stage 3 or stage 4 who underwent 23-gauge vitrectomy in Beijing Tongren Hospital from July 2015 to August 2018 were retrospectively enrolled. The exclusion criteria were as follows: stage 2 macular holes, traumatic macular holes, history of uveitis or other fundus diseases, and history of intravitreal injection, vitreous surgery or retinal detachment surgery. This study adhered to the tenets of the Declaration of Helsinki and was approved by the Ethics Committee of Beijing Tongren Hospital. Informed consent was obtained from all the subjects after explanation of the nature and possible consequences of the study.

Clinical charts of all patients were reviewed in detail, including date of birth (A), gender, affected eye, duration of symptoms (B), date of operation (C), intraocular pressure (IOP), axial length (AXL), and best-corrected visual acuity (BCVA). IOP came from noncontact tonometry (Full Auto Tonometer TX-F; Canon Canada, Quebec), and AXL was measured by IOL Master Biometry (Carl Zeiss Meditec, Jena, Germany). Every participant underwent slit-lamp microscopy examination for the anterior segment, and comprehensive fundus examinations were carried out by binocular indirect ophthalmoscope and fundus photography (fundus camera, TRC-50; Topcon, Tokyo, Japan). The diagnosis of IMH was based on clinical features, spectrum domain OCT (Cirrus high definition OCT; Carl Zeiss, Dublin, CA) imaging, fundus examination and intraoperative observation. According to Gass’s classification of 1995 [9], stages of IMH were identified by OCT and intraoperative observations, the majority of which were considered as large IMHs based on the 2013 consensus [10]. Age of onset was defined as the time when symptoms occurred and calculated by (C-A)-B. The recovery duration was the interval between the primary surgery and the occurrence of the highest BCVA during follow-up.

### OCT measurements

OCT examination was performed by the same experienced operator, and every measurement was repeated three times for an average to reduce manual errors. The entire macula was scanned by Macular Cube 512 × 128 protocol in horizontal and vertical orientations. The minimum linear diameter (MLD) was defined as the shortest distance between the broken ends of the neuroepithelia parallel to the retinal pigment epithelium (RPE). The basal diameter (BD) was the length of the RPE where the photoreceptors were detached, and H was the average vertical height on both sides, from the junction point of detached photoreceptor and RPE to the top of neuroepithelium. The fluid cuff (FC) was calculated by BD-MLD, diameter hole index (DHI) was MLD/BD, macular hole index (MHI) was H/BD, traction hole index (THI) was H/MLD, and hole form factor (HFF) was (MLD + BD)/BD.

Central macular thickness (CMT) was the automatic average thickness of foveal retina in a circular area with a diameter of 1 mm. The CMT of the affected eyes was undetectable, so CMT of the normal fellow eyes was measured to represent. Fellow eyes with normal CMT (within normal range of 240 ± 20 μm [11]), no history of fundus diseases or vitreous surgery and BCVA≥0.5 were included. Adjusted CMT was calculated by CMT/AXL.

### Surgical procedure

All the patients enrolled underwent standard 3-port pars plana vitrectomy by the same experienced surgeon using the 23-gauge technique. Phacoemulsification and intraocular lens implantation were combined prior to vitrectomy if necessary. After the core vitrectomy with the trocars inserted obliquely, the posterior cortical vitreous was detached (in stage 3 IMHs) and the vitreous was meticulously cut to the vitreous base. Then the internal limiting membrane (ILM) was peeled with intraocular forceps over a circle with a diameter of 2 to 3 optic discs centered in the macular hole without prior staining. Any epiretinal membranes would have been peeled before ILM peeling if presented. Afterwards, fluid in the hole was drained with the silicone-tipped flute needle and an adequate fluid-air exchange was performed. Gas or sterile air was respectively applied before and after July 2016, and the patients were asked to keep face-down position for 2 or 1 week respectively.

### Statistical analysis

All the clinical features, OCT parameters and surgical outcomes were compared between genders. BCVA was converted to logarithm of minimum angle of resolution (Log MAR) units. Statistical analysis was performed by SPSS 24.0 statistical software program (SPSS for Windows, Chicago, IL, USA). T-test was used for continuous variables when they obeyed normal distribution and Mann-Whitney U test was used when they did not. Pearson’s chi-square test and Fisher’s exact test were used for categorical variables. A *P* value less than 0.05 was considered to be statistically significant.

## Results

A total of 298 eyes from 280 patients (51 males and 229 females) with IMH of stage 3 (192 eyes) or stage 4 (106 eyes) were enrolled. The average age of all the patients was 64.7 ± 5.2 years old. The IMHs exhibited an average hole size of (620.5 ± 137.6) μm in MLD. The general primary closure rate was 92.3% (275/298), and the mean BCVA improved from 0.12 (Snellen 20/167) to 0.52 (Snellen 20/38) during a mean follow-up of (6.67 ± 4.33) months.

Comparing IMHs between genders of all the pre-operative characteristics, women exhibited a significantly higher ratio of stage3/stage4 (*P* = 0.045, Pearson’s chi-square test), higher IOP of the affected eye (*P* = 0.022, Mann-Whitney U test), and shorter AXL of both eyes (*P* = 0.001 for the affected eye, t-test; *P* < 0.001 for the fellow eye, Mann-Whitney U test) than men (Table 1), while other characteristics like age of onset and duration of symptoms showed no significant differences. For morphological parameters by OCT, women exhibited larger MLD (*P* = 0.009, t-test) in the horizontal orientation, but no difference in the vertical direction compared to men. Meanwhile, women demonstrated smaller H, MHI, and THI, but larger DHI and HFF in both orientations (Table 2). CMT (*P* < 0.001, Mann-Whitney U test) and adjusted CMT (*P* = 0.045, Mann-Whitney U test) of the fellow eyes were also significantly thinner in women than in men.

**Table 1:** Comparisons of clinical characteristics between genders

	Male	Female	P
No., patients/eyes	51/55	229/243	/
Affected eye, OD/OS	25/30	121/122	0.56
Bilaterality, bilateral/unilateral	4/47	14/215	0.75
Age of onset, years	65.2 ± 5.9	63.8 ± 5.1	0.17
Duration of symptoms, months	9.65 ± 1.42	9.29 ± 0.68	0.51
Stage, stage3/stage4	29/26	163/80	0.045 *
Pre-operative BCVA, Log MAR (Snellen)			
Affected eye	1.03 ± 0.33(20/167)	1.08 ± 0.39(20/167)	0.51
Fellow eye	0.21 ± 0.34(20/27)	0.19 ± 0.29(20/27)	0.74
IOP, mmHg			
Affected eye	14.5 ± 3.0	15.6 ± 3.2	0.022 *
Fellow eye	15.1 ± 3.4	15.3 ± 3.1	0.71
AXL, mm			
Affected eye	23.6 ± 0.7	23.2 ± 0.9	0.001 *
Fellow eye	23.7 ± 0.8	23.2 ± 1.0	< 0.001*
Combined cataract surgery, Yes/No	47/6	212/19	0.47
Tamponade, air/gas	35/18	133/98	0.26
Follow-up, months	5.63 ± 4.47	8.00 ± 7.07	0.08
Primary closure rate	96.2%	91.3%	0.39
Highest post-operative BCVA, Log MAR (Snellen)	0.34 ± 0.34(20/36)	0.38 ± 0.33 (20/39)	0.23
Recovery duration, months	4.72 ± 3.98	7.52 ± 7.33	0.024 *

*, *P* < 0.05; *BCVA* Best-corrected visual acuity, *Log MAR* Logarithm of minimum angle of resolution, *IOP* Intraocular pressure, *AXL* Axial length

**Table 2:** Comparisons of OCT parameters between genders

	Male	Female	P
Horizontal			
MLD, μm	577.1 ± 135.9	630.5 ± 136.3	0.009 *
BD, μm	1184.7 ± 233.8	1166.3 ± 210.6	0.94
H, μm	489.1 ± 84.6	458.3 ± 53.2	0.028 *
FC, μm	607.6 ± 180.4	535.9 ± 142.7	0.08
DHI	0.49 ± 0.09	0.54 ± 0.08	< 0.001 *
MHI	0.42 ± 0.07	0.40 ± 0.08	0.042 *
THI	0.90 ± 0.30	0.76 ± 0.21	0.001 *
HFF	1.49 ± 0.09	1.54 ± 0.08	< 0.001 *
Vertical			
MLD, μm	541.1 ± 132.9	578.5 ± 141.4	0.07
BD, μm	1089.8 ± 201.5	1088.1 ± 205.9	0.95
H, μm	485.4 ± 79.6	454.5 ± 52.7	0.012 *
FC, μm	548.7 ± 168.3	509.5 ± 136.7	0.07
DHI	0.50 ± 0.11	0.53 ± 0.09	0.016 *
MHI	0.46 ± 0.09	0.43 ± 0.09	0.043 *
THI	0.99 ± 0.49	0.84 ± 0.27	0.008 *
HFF	1.50 ± 0.11	1.53 ± 0.09	0.016 *
CMT (fellow eye), μm	247.6 ± 17.6	234.7 ± 13.4	< 0.001*
Adjusted CMT (CMT/AXL, fellow eye), μm/mm	10.5 ± 0.9	10.1 ± 0.7	0.045 *

*, *P* < 0.05; *OCT* Optical coherence tomography, *MLD* Minimum linear diameter, *BD* Basal diameter, *H* Height of hole, *FC* Fluid cuff, *DHI* Diameter hole index, *MHI* Macular hole index, *THI* Traction hole index, *HFF* Hole form factor, *CMT* Central macular thickness, *AXL* Axial length

As listed in Table 1, with comparable rate of combined cataract surgery and gas tamponade, the two genders exhibited similar primary closure rate (96.2% vs. 91.3%, *P* = 0.39, Fisher’s exact test) and highest BCVA (Snellen 20/36 vs. 20/39, *P* = 0.23, Mann-Whitney U test) within the comparable follow-up (*P* = 0.08, Mann-Whitney U test). However, men demonstrated significantly shorter recovery duration (*P* = 0.024, Mann-Whitney U test). Patients who underwent combined cataract surgery exhibited no significant difference in recovery duration between male and female (4.88 ± 4.00 vs. 7.16 ± 6.99, *P* = 0.11, Mann-Whitney U test), while in patients who did not undergo combined cataract surgery, the recovery duration of male was significantly shorter than that of female (3.40 ± 3.91 vs. 11.2 ± 9.60, *P* = 0.044, Mann-Whitney U test). Women who did not undergo combined cataract surgery exhibited significantly shorter recovery duration than those who did (7.16 ± 6.99 vs. 11.2 ± 9.60, *P* = 0.048, Mann-Whitney U test). However, men did not demonstrate the same difference (4.88 ± 4.00 vs. 3.40 ± 3.91, *P* = 0.34, Mann-Whitney U test).

Since the stage constituent ratio differed significantly between genders, comparisons of the main clinical features and OCT parameters were also conducted within stage respectively (Table 3). For IMHs of stage 3, women exhibited significantly younger age of onset (*P* = 0.023, Mann-Whitney U test), larger MLD (*P* = 0.003 in horizontal, *P* = 0.015 in vertical, both Mann-Whitney U test), and smaller H (*P* = 0.07, in horizontal, *P* = 0.029 in vertical, both Mann-Whitney U test) than men, while other clinical characteristics and surgical outcomes showed no difference. For IMHs of stage 4, all the pre-, intra-, and post-operative metrics exhibited no significant differences between genders.

**Table 3:** Comparisons between genders in stage 3 and stage 4 IMHs

	Stage 3 (*n* = 192)			Stage 4 (*n* = 106)		
	Male (*n* = 29)	Female (*n* = 163)	P	Male (*n* = 26)	Female (*n* = 80)	P
Age of onset, years	66.7 ± 6.7	63.6 ± 5.4	0.023 *	63.7 ± 4.6	64.7 ± 4.3	0.30
Duration of symptoms, months	6.79 ± 4.10	8.81 ± 10.10	0.56	12.6 ± 14.0	10.1 ± 10.7	0.18
Combined cataract surgery, Yes/No	27/2	143/13	1.00	20/4	69/6	0.20
Tamponade, air/gas	19/10	90/66	0.43	16/8	43/32	0.42
Follow-up, months	5.27 ± 3.85	6.84 ± 4.30	0.11	6.10 ± 5.23	7.11 ± 4.23	0.24
Primary closure rate	96.6%	91.0%	0.47	95.8%	92.0%	1.00
Recovery duration, months	4.62 ± 4.00	7.41 ± 7.29	0.07	4.86 ± 4.04	7.75 ± 7.46	0.17
Horizontal						
MLD, μm	563.5 ± 136.1	630.6 ± 126.1	0.003 *	592.4 ± 136.6	629.9 ± 156.1	0.28
BD, μm	1176.7 ± 248.0	1174.4 ± 204.4	0.49	1193.6 ± 221.5	1148.9 ± 223.3	0.38
H, μm	487.8 ± 80.4	457.3 ± 52.9	0.07	490.5 ± 90.6	459.7 ± 54.0	0.11
Vertical						
MLD, μm	520.3 ± 121.9	580.9 ± 136.1	0.015 *	564.2 ± 143.0	573.7 ± 152.4	0.78
BD, μm	1083.3 ± 210.1	1093.0 ± 200.1	0.81	1097.0 ± 195.3	1077.9 ± 218.2	0.69
H, μm	485.6 ± 74.9	452.4 ± 52.0	0.029 *	485.2 ± 86.0	458.7 ± 54.4	0.15

*, *P* < 0.05; *IMH* Idiopathic macular hole, *MLD* Minimum linear diameter, *BD* Basal diameter, *H* Height of hole

## Discussion

According to previous epidemiological investigations [3] and clinical reports [12], IMH is much more common to occur in women than in men. This study enrolled a population with a female to male ratio of about 4:1, which is higher than that of previous epidemiological investigations [3], but comparable to other retrospective clinical reports concerning surgical patients [13, 14]. The data presented here reveal that IMHs of women seem to be larger and occur earlier than those of men, but there are no significant differences between genders concerning other pre-operative metrics and surgical outcomes.

In this study, the stage constituent ratio (stage3/stage4) of IMHs was significantly higher in women than in men. According to Gass’s classification system [9], the difference between IMH of the two stages is mainly the status of the vitreous at the optic disc, so that women owned more IMHs of stage 3 indicated the possibility that complete posterior vitreous detachment (PVD) occurs later in women than in men. The study of Schwab and colleagues observed 335 non-myopic eyes and found that women were significantly older than men when having the late-stage PVD (complete PVD) in the eyes [15], which was consistent with our speculation. This phenomenon could be explained by the findings of van Deemter and colleagues that female vitreous, especially after 50 years of age, experiences a faster accumulation of pentosidine [16], which is associated with the absence of a complete PVD [17].

Kazuyuki et al. observed 526 eyes of 480 patients with stage 3 or stage 4 IMHs that had undergone vitrectomy, and found that females of stage 3 demonstrated younger age of onset and larger size of macular hole [18]. Our results seem to be in agreement with this. However, the report of Kazuyuki et al.’s was not in English and the full text was not available online, so we cannot obtain any further details.

In high myopic eyes, macular holes occur earlier [19] and evolve faster than in non-myopic eyes [20] due to longer axial length [20] and thinner retina thickness [21] because these two factors may exacerbate the impact of traction forces on highly myopic eyes [22].

In the present study, the foveal retina thickness of eyes with IMH was unable to know owing to the hole, so CMT of the fellow eye was measured to represent. The results showed that AXL was longer and CMT was thicker in male than in female, which were in accordance with previous reports [23, 24] but seemed to be a contradiction concerning their opposite effects on development of macular holes. Meanwhile, CMT is positively related to AXL [24]. Therefore, we introduced the concept of adjusted CMT calculated by CMT/AXL, regarding CMT and AXL as a whole, and found it significantly smaller in female than in male. This could probably explain why female exhibited younger age of onset in IMHs of stage 3. In other words, though developing stage 3 IMH earlier, women tend to remain in the same stage longer than men, and this also explains why IMHs of stage 4 showed no significant difference between genders in age of onset in our results.

With respect to morphological metrics, female IMHs exhibited larger MLD than male on the whole, which largely attribute to the difference in stage 3 IMHs. According to previous studies, MLD enlarges as the IMH progresses, while enlargement of BD slows down [25]. Since female develop stage 3 IMHs earlier and stay in the same stage longer, it is reasonable that female IMHs of stage 3 are larger in MLD than male. As the disease evolves, the difference of MLD became much less prominent between genders in stage 4 IMHs, which indicates that IMHs of both male and female evolve into a similar ultimate state.

The height of the hole was smaller in women than in men. Previous reports gave different conclusions on whether height of macular hole could predict surgical outcomes [26, 27]; however, their definition of the height - the greatest distance between the RPE layer and the vitreoretinal interface- differed from ours. In the present study, height of the hole was defined as the vertical height of the neuroepithelia edge starting from the junction point of the detached photoreceptor and the RPE, which we believe to be in accordance more with retinal thickness than retinal edema. In this regard, smaller H represents a relatively thinner foveal retina, which is consistent with the normal variation that CMT is smaller in women than in men.

Other derived indexes were calculated by MLD, BD, and H, so they showed understandable differences between genders in accordance with the three primary parameters.

According to previous studies, primary closure rate is mainly correlated with MLD and duration of symptoms, and post-operative BCVA with MLD and times of surgery [28]. In this study, men and women showed similar primary closure rate and comparable highest BCVA during follow-up. That is to say, the differences between genders mentioned above may largely attribute to normal variations in foveal anatomy and physiology, but demonstrate limited influence on surgical outcomes; in other words, female IMHs may be not more severe than male as it may be anticipated. Our results showed that women who did not undergo combined cataract surgery demonstrated significantly longer recovery duration, which resulted in that women exhibited longer recovery duration than men in general. The larger MLD and the thinner adjusted CMT of female could be one of the causes, but further elucidation is in need. Besides, the ellipsoid zone and the external limiting membrane could regenerate slower in eyes with larger MLD, which may also affect the recovery duration.

This retrospective study enrolled only patients who underwent operation, which may lead to a sample selection bias; however, the relatively large size of sample with rational gender proportion and rigorous methods could offer reliable results to a large extent. Usually, stage 2 IMHs are asymmetric in most scan directions because of the vitreomacular traction, which may lead to inaccuracy of parameter measurements like MLD and H. Thus, IMHs of stage 2 were excluded in this study.

## Conclusion

This study comprehensively compares the gender-related differences in IMHs of stage 3 and stage 4 with a relatively large size of sample, which may enrich perception of IMHs and provide references for further research. Though women seem to develop IMHs earlier and larger, these differences could be explained by the normal anatomical and physiological variations between genders to a certain extent, and may not considerably influence the surgical outcomes.

## Data Availability

All data relevant to the study are included in the article. The deidentified participant data are in the team’s database, which is available only from the corresponding author. For reusing, please contact WL (wuliubj@sina.com) for permission.

## Abbreviations

IMH: Idiopathic macular hole
CSC: Central serous chorioretinopathy
IOP: Intraocular pressure
AXL: Axial length
BCVA: Best-corrected visual acuity
MLD: Minimum linear diameter
RPE: Retinal pigment epithelium
BD: Basal diameter
H: Height
FC: Fluid cuff
DHI: Diameter hole index
MHI: Macular hole index
THI: Traction hole index
HFF: Hole form factor
CMT: Central macular thickness
ILM: Internal limiting membrane
Log MAR: Logarithm of minimum angle of resolution
PVD: Posterior vitreous detachment
